# Liver transplantation for homozygous familial hypercholesterolaemia

**DOI:** 10.1097/MOL.0000000000001007

**Published:** 2025-09-10

**Authors:** Gilbert R. Thompson, Shahenaz Walji, Jaimini Cegla

**Affiliations:** a Division of Diabetes, Endocrinology and Metabolism, Imperial College London; b Lipids and cardiovascular risk service, Department of Cardiology, Hammersmith Hospital, Imperial College Healthcare NHS Trust, London, UK

**Keywords:** cardiovascular, drug therapy, liver

## Abstract

**Purpose of review:**

The review focusses on the role of liver transplantation, and rarely combined liver and heart transplantation, in the current management of homozygous familial hypercholesterolaemia (HoFH).

**Recent findings:**

The review features world-wide reports published during the past 10 years describing the rationale and outcomes of liver transplantation for children and adults with HoFH. It also provides information on the scale of liver and heart transplantation for a variety of other disorders.

**Summary:**

Liver transplantation provides a more effective means of lowering LDL than currently available alternatives such as apheresis and lomitapide but carries with it an unacceptably high risk of posttransplant morbidity and mortality. This is mainly due to the adverse effects of life-long immunosuppressive drug therapy, which restricts the use of liver transplantation to those HoFH patients in whom optimal medical therapy has failed.

## INTRODUCTION

The first liver transplant for homozygous familial hypercholesterolaemia (HoFH) was performed in Pittsburgh, USA by Starzl *et al.* [1]. The patient was a 6 year-old girl with HoFH and a serum cholesterol of 31 mmol/l who was undergoing metabolic studies at the University of Texas Southwestern Medical School in Dallas. While there, the patient developed recurrent bouts of angina and heart failure and underwent a coronary bypass and replacement of her mitral valve. Despite this her cardiac condition was so poor that liver transplantation was considered too dangerous unless accompanied by a concomitant heart transplant. This dual procedure was carried out successfully during a 16-h operation at the Children's Hospital and University Health Center of Pittsburgh in 1984 [2]. 

**Box 1:**
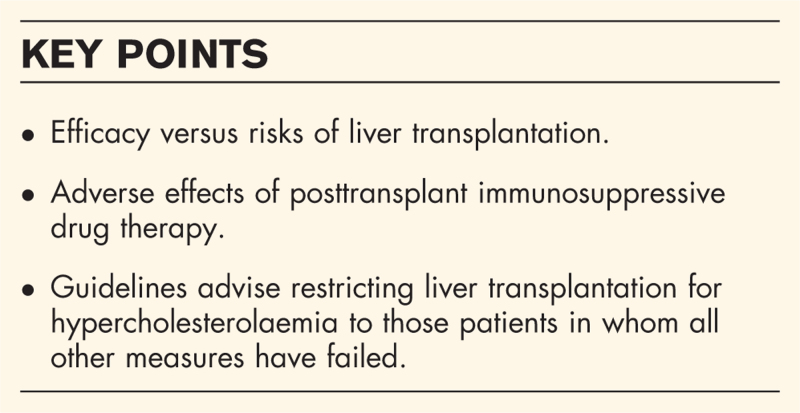
no caption available

**FIGURE 1:**
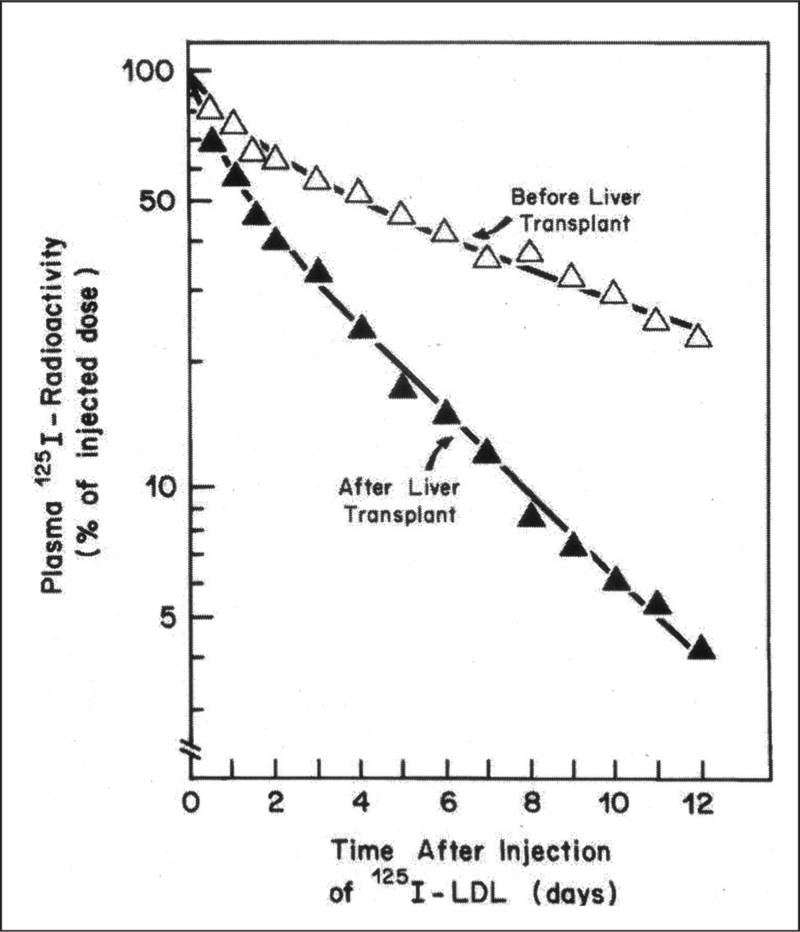
Turnover of ^125^I -labelled LDL in HoFH patient before and after liver transplantation (reproduced from Bilheimer DW, Goldstein JL, Grundy SM, Starzl TE, Brown MS. Liver transplantation to provide low-density-lipoprotein receptors and lower plasma cholesterol in a child with homozygous familial hypercholesterolemia. N Engl J Med. 1984; 311:1658–64).

Subsequently the patient was transferred back to Dallas for further studies of LDL metabolism by Bilheimer *et al.* [3]. These studies showed that the fractional catabolic rate of ^125^ I- LDL had increased 2.5-fold, from 0.12 pools/day preoperatively to 0.31 pools/day postoperatively, confirming the absence of hepatic LDL receptors in HoFH and their presence in the transplanted normal liver (Fig. 1). The increased rate of removal of LDL posttransplant resulted in an 80% decrease in the patient's serum LDL cholesterol from 25 mmo/l to 4.8 mmol/l. The patient was discharged from hospital one month after her transplants, taking prednisone and ciclosporin for immunosuppression. She died seven years later, aged 13 [4].

This remarkable medico-surgical *tour de force* established the scientific basis for performing liver transplantation in HoFH and demonstrated the feasibility of combining this procedure with a heart transplant in patients with end stage cardiac disease. The current review describes the pros and cons of liver transplantation, with or without concomitant heart transplantation, and compares it with nonsurgical lipid-lowering therapy to treat HoFH.

## THE CARDIOVASCULAR CONSEQUENCES OF HYPERCHOLESTEROLAEMIA

A retrospective survey of 44 HoFH patients in the UK analyzed the frequency of cardiovascular (CV) events during a 50 year follow up period between 1979 and 2014 [5]. Many of the patients sustained multiple events. Overall, aortic stenosis occurred in 48%, aortic valve replacement was performed in 23%, coronary artery disease occurred in 58% and coronary artery bypass grafting (CABG) was undertaken in 30%. These CV events occurred significantly more often in the 30% of patients who died. Baseline serum cholesterol levels were similar in patients still alive (20.2 mmol/l) and in those who died (21.3 mmol/l) but the on-treatment level was much lower in the former (8.1 mmol/l) than in the latter (14.5 mmol/l), reflecting improvements in lipid-lowering therapy from 1990 onwards.

This study illustrated both the frequency and severity of the adverse cardiovascular consequences of HoFH and their dependence upon the on- treatment level of serum cholesterol.

## SEARCH STRATEGY AND SELECTION CRITERIA

Sources of information for the manuscript included review articles and guidelines by international scientific organizations, supplemented by searches of PubMed for peer-reviewed, full-length original research articles in all languages Search terms were combinations of the following terms: “homozygous familial hypercholesterolemia”, “liver transplantation”, “heart transplantation” and “immunosuppressive drugs.”

## ROLE OF LIVER TRANSPLANTATION IN HYPERCHOLESTEROLAEMIA

Table 1 lists details of publications reporting five or more cases of liver transplantation for HoFH during the past 10 years, the largest series being that of Mansoorian *et al.* in Iran [6]. There the diagnosis of HoFH was based on clinical not genetic criteria and 20% of the patients were considered to be heterozygotes. Ages at transplantation ranged from 2.5–10 years and most patients achieved a posttransplant LDL cholesterol of < 5.2 mmol/l. Thirty-six patients were followed up for 0.5–6 years, during which time there were 3 deaths. The other case series reported in Table 1 were from Turkey [7,8], USA [9], Saudi Arabia [10,11^▪^], China [12–14], and Australia and New Zealand [15].

**Table 1:** Reports of five or more liver transplants for homozygous FH, 2015–2025

Reference	n	Age at Tx	LDL-C mmol/l		Follow up	Deaths
		Years	Pre-Tx	Post-Tx	years	*n*
Mansoorian [6]	36^a^	5–28	>13 (*n* = 24) 7.8–13 (*n* = 12)	<3.4 (*n* = 14) 3.4–5.2 (*n* = 16) >5.2 (*n* = 3)	0.5–6	3
Alim [7]	8	6-12	14.9	3.0	0.3–8	2
Martinez [9]	8	2-17	15.6	2.7	2–6	0
Al Dubayee [10]	9	6-18	20.7	5.1	0.5–28	3
Chen [12]	5	6–22	17.1	3.0	2–2.8	0
Page [15]	9	3–26	11.0	2.6	4–27	1
Al- Ashwal [11^▪^]	17	0.8–3	20.7	3.3	8–24	0
Dagli [8]	5	4–10	22.4	3.6	1.5–16	2
Lin [13]	14	2–12	13.9	2.4	2,3–5.9	0
Zhan [14]	6	3.5–12	12.3	2.5	1.6–7.8	0
	117	0.8–28	16.5 ± 4.0^b^	3.1 ± 0.8^b^	0.3–28	11^c^
				- 81%		9.4%

a 20% were probably HeFH.

b Mean ± SD excluding Mansoorian [6].

c Seven deaths were cardiovascular, 4 were transplant-related.

Altogether, a total of 117 patients aged 0.8–28 years (several being below the age of 3) underwent liver transplants. Pretransplant LDL cholesterol (mean ± SD) was 16.6 ± 4.0 mmol/l which decreased by 81% to 3.1 ± 0.8 mmol/l posttransplant. Follow up ranged from 0.3–28 years, during which there were 11 deaths (9.4%). 7 deaths were due to cardiovascular disease, 4 were transplant related. Hence, on the basis of these data one may conclude that liver transplantation is a highly effective means of lowering LDL in HoFH patients but carries with it a roughly 10% risk of death.

Not all authors agree that liver transplantation is sufficiently effective in lowering LDL in HoFH. Al Dubayee *et al.* [10] concluded that it did not enable attainment of guideline LDL cholesterol targets and most of their multinational patients required posttransplant lipid-lowering therapy. Similarly, Dagli *et al.* [8] found that liver transplantation did not lower LDL cholesterol enough to prevent the subsequent development of cardiovascular disease in their Turkish homozygotes. Notably, several of the deaths in Table 1 were in Turkish patients.

## COMBINED LIVER AND HEART TRANSPLANTATION IN HYPERCHOLESTEROLAEMIA

Far fewer combined heart-liver transplants for HoFH have been reported in the literature than reports of liver transplants, as listed in Table 2. This reflects the fact that the combined procedure is technically more difficult and is usually carried out only in desperate circumstances, exemplified by the young girl operated upon in Pittsburgh in 1984 [1]. A search of PubMed revealed a further seven cases published up to 2012 but none since. Three case reports were from the USA [1,2,16], two from the UK [17,18], two from Spain [19,20] and one from Norway [21].

**Table 2:** Combined heart and liver transplantation for homozygous FH, 1984–2012

Reference	*n*	Age at Tx	TC mmol/l^a^		Follow up	Outcome
		Years	Pre-Tx	Post-Tx	years	
Starzl [1]						
Bilheimer [3]	1	6	27.9	7.8	7	Died aged 13
Giakoustidis [4]						
Shaw [2]	1	17	–	–	0	Died postop
Fricker [16]	1	6	–	–	2	Alive and well
Valdivielso [19]	1	12	19.0	5.4	1.5	Alive and well
Barbir [17]	1	33	23.0	5.6	20	Alive and well
Ibrahim [22]						
Rela [18]	1	33	–	–	3.7	Alive and well
–						
De Peralta [20]	1	9	–	–	9	Alive and well
Offstad [21]	1	46	25.0	4.5	4	Alive and well
	8	6–46	23.7	5.8	0–20	2 deaths
				−76%		25%

a TC, Serum total cholesterol.

Examination of the data in Table 2 shows that the age of HoFH patients at the time of surgery ranged from 6 to 46 years. Some of the reports provide details of changes in serum total cholesterol but not LDL cholesterol while others contain no information on serum lipids. In those reports that do, the mean serum cholesterol was 23.7 mmol/l preoperatively, which decreased by 76% to 5.8 mmol/l postoperatively. Follow up ranged from 0 to 20 years, during which there were 2 deaths (20%).

One of the most remarkable of these cases was the 33 year old female patient described by Barbir *et al.* [17] and later by Ibrahim et [22] who had severe coronary disease, aortic stenosis and heart failure at the time of her combined transplant but was alive and well 20 years afterwards. This case illustrates that combined heart-liver transplantation is a life-saving procedure for HoFH patients with end stage cardiac disease, which can quite literally result in their cardio-metabolic rebirth by substituting a liver with LDL receptors for one lacking them and substituting a normal heart for one with severe atherosclerosis. Another potential benefit of a heart - liver transplant is the evidence that combined organ transplantation affords some degree of protection against rejection [22].

## TRANSPLANT REGISTRY DATA

Data from the Organ Procurement and Transplantation Network (OPTN) and the Scientific Registry of Transplant Recipients (SRTR) indicate that >10 000 liver transplants were performed in the USA in 2023, 95% of which were in adults [23^▪^]. The commonest indication for a liver transplant was alcohol-related liver disease. Most transplants (58%) were performed within a year of the recipient being added to the waiting list. The majority of transplant recipients were treated with a combination of tacrolimus, a mycophenolate agent and a steroid. Graft failure occurred in 20.7% of recipients within 5 years and there was a similar 5-year mortality.

The commonest diagnosis among pediatric recipients of a liver transplant was biliary atresia (37.5%) and 25% of such transplants were undertaken in children < I year old. A minority of liver transplants (15%) were for metabolic disorders, which presumably included HoFH.

Data from the European Transplant Registry (ELTR) show that 1- and 5- year postliver transplant survival rates were 80–84% and 65–75% respectively and were inversely related to age [24]. Data from yet another registry, the United Network for Organ Sharing (UNOS), showed that over 40% of deaths occurred within the first year post transplant, predominantly from infection and cardiovascular causes, whereas deaths from cancer tended to occur later [25]. Deaths from transplant failure or rejection were relatively infrequent.

The number of heart transplants performed in the USA in 2023 was 4599, 89% of which were in adults [26^▪^]. Among patients placed on the waiting list in 2022, 58% were transplanted within three months and 71% within one year. The commonest reason for a heart transplant in adults was cardiomyopathy (62%) followed by coronary artery disease (28%). In children the commonest cause was congenital heart disease (65%) followed by Other/Unknown (18%). Five- year posttransplant survival was 80% in adult and 84% in pediatric recipients. Combined heart – liver transplants were performed in 1.7% of adult recipients and in 0.6% of paediatric recipients, with a 5-year survival of 82%. Immunosuppressive therapy for heart transplants was similar to that used for liver transplantation.

Although only a tiny minority of transplants are for HoFH, the registry data provide useful information regarding the risks and outcomes of transplanting livers and hearts for a variety of disorders. Many of the risks arise from the need for posttransplant immunosuppressive drug therapy.

## IMMUNOSUPPRESSIVE DRUG-RELATED ADVERSE EFFECTS

Medical or nontechnical issues are the leading cause of morbidity and mortality posttransplant, mainly due to the adverse effects of long-term immunosuppression. Since the majority of liver transplant recipients require lifelong immunosuppression, it is imperative that immunosuppressive regimens be tailored to the lowest dose required to prevent allograft rejection [27].

Calcineurin inhibitors, particularly tacrolimus are the cornerstone of maintenance immunosuppression after liver transplantation [28]. The most frequent and important side-effects of calcineurin inhibitors are nephrotoxicity, posttransplant diabetes mellitus, hypertension and dyslipidaemia. Diabetes mellitus occurs in 15%–45% of postliver transplant patients. These side effects can be mitigated by reducing tacrolimus exposure by combining it with other immunosuppressants, namely antiproliferative agents (mycophenolic acid, azathioprine) and mammalian target of Rapamycin (mTOR) inhibitors (everolimus, sirolimus). Steroids may be necessary to combat T cell- mediated rejection [29]. Immunosuppression may also cause leucopoenia, which increases the risk of death from infections in the first year post transplant [25].

The important side effects of immunosuppressive drug therapy post liver transplant are summarised in Table 3.

**Table 3:** Side effects of post liver transplant immunosuppressive therapy (adapted from Noble *et al.* [28])

Nephrotoxicity	More common with ciclosporin and in combination with mTOR inhibitors
NODAT (New Onset Diabetes after transplant) or PTDM (Post Transplant Diabetes mellitus)	Diagnosis based on 2003 WHO and ADA criteria occurs in 15%–45% of liver transplant patients from Year 1–5. More frequently seen with Tacrolimus. Steroids can also induce Diabetes
Hypertension	Sustained hypertension in 35–55% of patients. More frequently seen with Ciclosporin
Dyslipidaemia	Present in 40–66% of liver transplant recipients. Calcineurin and mTOR inhibitors and corticosteroids increase the risk
Increased CVD risk	All immunosuppressants
Metabolic syndrome, dyslipidaemia, osteoporosis, avascular necrosis, poor wound healing, psychosis, adrenal suppression	Corticosteroids
De novo malignancy development [25]	Liver transplant recipients are at 2x–3x increased risk

## COMPARISON OF LIVER TRANSPLANTATION WITH OTHER LIPID-LOWERING THERAPIES FOR HYPERCHOLESTEROLAEMIA

**FIGURE 2:**
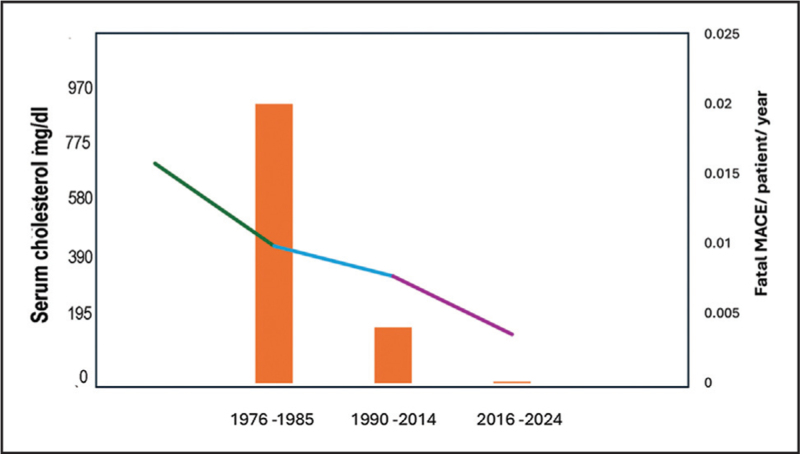
Temporal improvements in cholesterol- lowering efficacy were associated with a reduction in fatal major adverse cardiovascular events in HoFH (reproduced from Cegla J, Walji S, Barton L, Neuwirth C, Thompson GR. Treatment of Homozygous Familial Hypercholesterolemia. JACC Adv. 2025;4:101708. doi:10.1016/j.jacadv.2025.101708. Epub 2025 Apr 26. PMID: 40288084; PMCID: PMC12059681).

In a recent state of the art review of nonsurgical lipid-lowering therapy for HoFH, the authors analyzed the data gathered from treating patients with this disorder referred to Hammersmith Hospital, London since 1976 [30^▪^]. The best results were obtained between 2016–2024 when lipoprotein apheresis was combined with a statin, ezetimibe and lomitapide, which reduced serum cholesterol by 46% from 7.8 to 4.2 mmol/l (Fig. 2).

Confirmation of the benefits of effective cholesterol-lowering therapy in reducing CVD events and mortality came from a larger survey of South African and UK homozygotes [31]. That study provided further evidence that the extent of reduction of serum cholesterol, achieved in this instance by a combination of statins, ezetimibe, lipoprotein apheresis, and evolocumab, is a major determinant of survival in HoFH.

A recently published study compared the lipid-lowering effects of nonsurgical treatment and liver transplantation in in a large cohort of genetically- confirmed HoFH patients in Saudi Arabia [32^▪▪^]. Of the 88 homozygotes, 75% were the result of consanguineous unions. Most of those who underwent liver transplantation were described in an earlier publication [11^▪^].

Comparison of lipid-lowering therapy in this cohort of HoFH patients showed that lipoprotein apheresis plus statin and ezetimibe reduced LDL cholesterol by 55%, lomitapide plus statin and ezetimibe by 69% and liver transplantation by 83%. Although liver transplantation was the most effective means of lowering LDL, 17% of fatalities were transplant related. Furthermore, severe aortic valve stenosis has been shown to progress in an HoFH patient after a liver transplant despite normalization of lipid levels [33]; this phenomenon has also been reported after treatment with lipoprotein apheresis [34]. The best way to prevent this unintended consequence of treatment is to prevent aortic root atheroma from developing in the first place, by early diagnosis of HoFH and instituting radical lipid-lowering therapy as soon as possible in childhood [35].

Recently, evinacumab, a monoclonal antibody that inhibits angiopoietin-like-3 (ANGPTL3), was shown to lower LDL-C in 12 homozygotes by 56% from 6.5 to 2.8 mmol/l when given intravenously every 4 weeks. None of the evinacumab-treated patients, 10 of whom were on lipoprotein apheresis, experienced a cardiovascular event over a 4-year period, compared with 24% of a control cohort matched for apheresis but not on evinacumab [36]. The LDL level in patients on evinacumab in this study was comparable with the postliver transplant levels in Table 1. The drug has even been used to successfully treat a 13-month-old child with HoFH without any side effects, in contrast with his older sibling who had earlier suffered a fraught postoperative period after a liver transplant [37]. That said, evinacumab may often prove to be an adjunct to apheresis rather than a substitute [38].

Finally, there is the prospect of in vivo gene editing employing CRISPR (clustered regularly interspaced short palindromic repeats) base-editors. Verve Therapeutics are currently developing a CRISPr base-editing medication targeting ANGPLT3 (Verve-201) which, if proven to be safe and effective, might be a life-changing advance in the treatment of HoFH.

## GUIDELINE STATEMENTS ON LIVER TRANSPLANTATION FOR HYPERCHOLESTEROLAEMIA

Clinical guidelines recognize liver transplantation as a potential treatment option for HoFH in select cases, though it is generally considered a last resort.

According to the 2023 European Atherosclerosis Society (EAS) consensus statement, liver transplantation may be an option for a limited number of patients with homozygous familial hypercholesterolemia, especially severely affected young children with bi-allelic null mutations [39]. According to the EAS, liver transplantation should be considered only as a final measure when intensive lipid-lowering treatments have proven ineffective. It may be appropriate in cases of progressive coronary artery disease with LDL-C levels above 1.8 mmol/l (70 mg/dl), or in patients with minimal or stable coronary disease but persistently high LDL-C levels exceeding 13 mmol/l (500 mg/dl). In instances of rapidly advancing disease or extensive cardiac involvement, combined liver and heart transplantation may be warranted. Nevertheless, it also recognizes that liver transplantation is associated with considerable risks, including surgical complications, the possibility of both acute and chronic graft rejection, and the adverse effects of long-term immunosuppressive therapy. Optimal outcomes depend on access to experienced pediatric or adult transplant centres and careful assessment of the patient's ability to adhere to lifelong medical care. The EAS panel recommends establishing a global registry for patients undergoing liver transplantation for HoFH to support better evaluation and understanding of its long-term safety and effectiveness.

Similarly, other international and national clinical guidelines, including those from the International Atherosclerosis Society [40], National Lipid Association [41], Heart UK [42] and FH Australasia [43] consider liver transplantation for HoFH for the most severe of cases only after all other treatment options have been exhausted.

In countries where the shortage of whole liver donors is critical, an alternative approach is to undertake a partial liver transplant from a living relative with heterozygous FH. This is regarded as a valid therapeutic option in Japan but further studies and clinical experience are required to establish it as a safe and effective treatment for HoFH [44].

## CONCLUSION

Any form of radical therapy for a life-threatening disorder needs to be rigorously assessed both in terms of its efficacy and safety. Published data suggest that liver transplantation is the single most effective means of lowering LDL cholesterol in adults and children with HoFH. When combined with heart transplantation it offers the HoFH recipient a cardio-metabolic rebirth. However, these advantages are more than offset by the risks of transplantation, which stem largely from the need for life-long immunosuppressive therapy and result in an unacceptably high rate of posttransplant mortality. Hence, in keeping with current national and international guidelines, liver transplantation for HoFH should be resorted to only after all other medical options, including apheresis and maximally tolerated pharmacotherapy, have failed.

(1) This review describes the recent literature reporting the efficacy and outcome of liver transplantation, occasionally combined with heart transplantation, for HoFH.
(2) It compares the effectiveness of liver transplantation with medical forms of LDL-lowering therapy.
(3) The data summarised in the review support the opinion of national and international guidelines that liver transplantation should be used only as a last resort to treat HoFH.

## Acknowledgements

*None.*

### Financial support and sponsorship

*None.*

### Conflicts of interest

*J.C. reports talks, consultancies, or research funding from Amgen, Sanofi, Daiichi Sankyo, Amryt, Novartis, Akcea, Ultragenyx, Chiesi, Eli Lilly, Menarini and Verve Therapeutics. All other authors have reported that they have no relationships relevant to the contents of this paper to disclose.*

## REFERENCES AND RECOMMENDED READING

Papers of particular interest, published within the annual period of review, have been highlighted as:

▪ of special interest
▪▪ of outstanding interest
